# Selective Inhibition of Heparan Sulphate and Not Chondroitin Sulphate Biosynthesis by a Small, Soluble Competitive Inhibitor

**DOI:** 10.3390/ijms22136988

**Published:** 2021-06-29

**Authors:** Marissa L. Maciej-Hulme, Eamon Dubaissi, Chun Shao, Joseph Zaia, Enrique Amaya, Sabine L. Flitsch, Catherine L. R. Merry

**Affiliations:** 1 Materials Science Centre, School of Materials, The University of Manchester, Grosvenor St., Manchester M1 7HS, UK; 2 Division of Cell Matrix Biology & Regenerative Medicine, Faculty of Biology, Medicine and Health, Michael Smith Building, The University of Manchester, Oxford Road, Manchester M13 9PT, UK; eamon.dubaissi@manchester.ac.uk (E.D.); enrique.amaya@manchester.ac.uk (E.A.); 3 Center for Biomedical Mass Spectrometry, Boston University School of Medicine, 670 Albany Street, Boston, MA 02118, USA; Chun.Shao@bms.com (C.S.); jzaia@bu.edu (J.Z.); 4 School of Chemistry & Manchester Institute of Biotechnology, The University of Manchester, 131 Princess Street, Manchester M1 7DN, UK; Sabine.Flitsch@manchester.ac.uk

**Keywords:** heparan sulfate, azido sugar, glycosaminoglycan, carbohydrate biosynthesis, small molecule inhibitor, biorthogonal chemistry

## Abstract

The glycosaminoglycan, heparan sulphate (HS), orchestrates many developmental processes. Yet its biological role has not yet fully been elucidated. Small molecule chemical inhibitors can be used to perturb HS function and these compounds provide cheap alternatives to genetic manipulation methods. However, existing chemical inhibition methods for HS also interfere with chondroitin sulphate (CS), complicating data interpretation of HS function. Herein, a simple method for the selective inhibition of HS biosynthesis is described. Using endogenous metabolic sugar pathways, Ac_4_GalNAz produces UDP-GlcNAz, which can target HS synthesis. Cell treatment with Ac_4_GalNAz resulted in defective chain elongation of the polymer and decreased HS expression. Conversely, no adverse effect on CS production was observed. The inhibition was transient and dose-dependent, affording rescue of HS expression after removal of the unnatural azido sugar. The utility of inhibition is demonstrated in cell culture and in whole organisms, demonstrating that this small molecule can be used as a tool for HS inhibition in biological systems.

## 1. Introduction

Heparan sulphate (HS) is a prevalent glycosaminoglycan (GAG) attached to protein cores (proteoglycans) on the cell surface of almost every cell type. HS proteoglycans form an integral part of the extracellular matrix with important roles in development [1], homeostasis [2,3] and disease [4,5]. HS is involved in cell-cell and cell-matrix communication, fine-tuning cellular responses to the extracellular milieu.

HS biosynthesis consists of a repeating disaccharide unit structure of glucuronic acid–*N*-acetylglucosamine (GlcA-GlcNAc) polymerised by the exostoses enzyme complex (EXT1/2) from UDP-GlcA and UDP-GlcNAc active nucleotide donor sugars [6,7,8]. During this process the *N*-deacetylase/*N*-sulphotransferase (NDST) enzymes work in tandem to begin modification of the nascent chain. The NDST enzymes can replace the acetyl group on GlcNAc with a sulphate [9], often providing the gateway step for further modifications of the chain. Additionally, the NDSTs are also involved in control of HS chain length [10] with NDST shown to be co-localised with EXT2 [11]. During extension of the backbone, several other chemical modifications are possible, resulting in fine patterning of the chain where the functionality of HS is encoded. O-sulphotransferases (OSTs) modify the HS chain at the 2-, 6- and 3-O position or epimerisation of GlcA to iduronic acid (IdoA) by C5-epimerase can occur. Together, these enzymes contribute to HS functionality by influencing the fine patterning of the chain [12].

Despite its widespread role in biology, few chemical tools exist for the manipulation of HS function, with those available often interfering with chondroitin sulphate/dermatan sulphate (CS/DS) pathways simultaneously. Methods to ablate HS exist via targeted genetic deletion of biosynthetic HS enzymes [6,7]. However, genetic manipulation is costly and labour intensive with embryonic lethality in null mutant animals [7], posing challenges for post-embryonic analysis. In contrast, chemical approaches offer cheap, user-friendly alternatives, which either perturb sulphation of the chain [13,14] or compete with endogenous substrates involved in HS assembly, such as amino sugar derivatives [15,16] and mimics of tetrasaccharide linkages [17,18,19,20]. However, the additional effect on CS/DS synthesis can complicate data interpretation particularly when both GAGs are displayed on the proteoglycan of interest [21].

**Figure 1:**
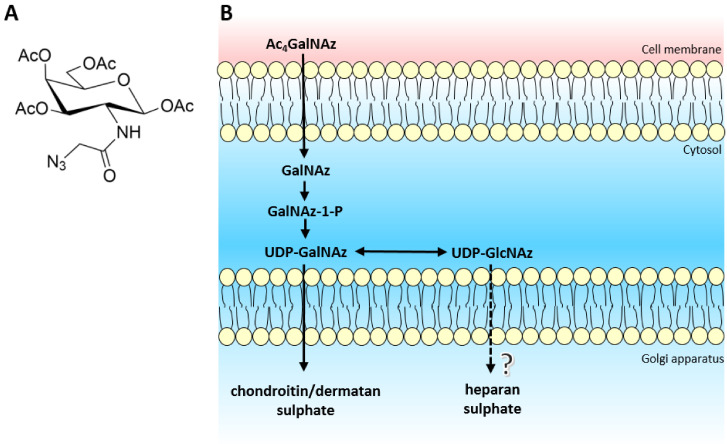
(**A**) Structure of tetra-acetylated *N*-azidoacetylgalactosamine. (**B**) Schematic of biological azido sugar precursor production for GAG synthesis. Ac_4_GalNAz travels across the cell membrane and enters the cytoplasm. Endogenous deacetylases remove the acetyl protective groups leaving GalNAz, ready to enter the GalNAc salvage pathway. After a cascade of enzymes, both UDP-GalNAz and UDP-GlcNAz are produced, which target CS/DS and potentially HS biosynthesis respectively.

Azido sugars and other bio-orthogonal chemistry approaches have been demonstrated as useful functionalised chemical probes to label *N*-glycans [22], O-GlcNAc modifications [23], mucin type O-GalNAc glycans [24] and sialic acid moieties [25]. Tetra-acetylated *N*-azidoacetylgalactosamine (Ac_4_GalNAz) can be metabolically converted to UDP-GlcNAz and UDP-GalNAz via the GalNAc salvage pathway [23], potentiating its use in GAG synthesis (Figure 1).

The azido sugar nucleotide donors mimic UDP-GalNAc and UDP-GlcNAc, required for CS/DS and HS biosynthesis respectively. Recently, the EXT1/2 enzyme complex has been shown to utilise UDP-GlcNAz as a substrate for the addition of GlcNAz to the non-reducing termini of heparan sulphate chains in vitro [26]. However *in vivo*, there is the potential that UDP-GlcNAz could interfere with the interaction or activity of the HS polymerisation machinery (EXT/NDST enzymes) due to the location of the azido group situated on the acetyl position of the GlcNAc residue, thereby producing an inhibitory effect on the biosynthetic pathway. Therefore, we sought to extend and validate the use of Ac_4_GalNAz treatment as a potential novel, small chemical inhibitor of HS synthesis.

## 2. Results and Discussion

**Figure 2:**
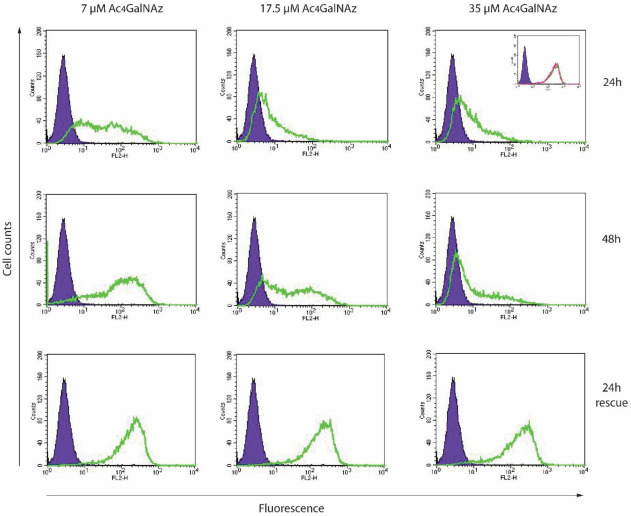
Flow cytometric analysis of Ac_4_GalNAz-treated CHOs. Cells were treated with 7–35 µM Ac_4_GalNAz for 24–48 h, or for the first 24 h, then 24 h without Ac_4_GalNAz (24 h rescue) and analysed for cell surface anti-HS (10E4) reactivity. Purple infilled, antibody control. Green trace, Ac_4_GalNAz-treated cells. Inset, experimental controls: purple infilled, antibody control; green trace, vehicle-treated; pink trace, untreated.

Chinese hamster ovary (CHO) cells were treated with different concentrations (7–35 µM) of Ac_4_GalNAz. No lethal effect on the cells was observed. Cell surface HS was analysed using flow cytometry. A reduction in anti-(10E4) HS antibody staining was observed at the cell surface in response to increasing Ac_4_GalNAz concentration (Figure 2).

**Figure 3:**
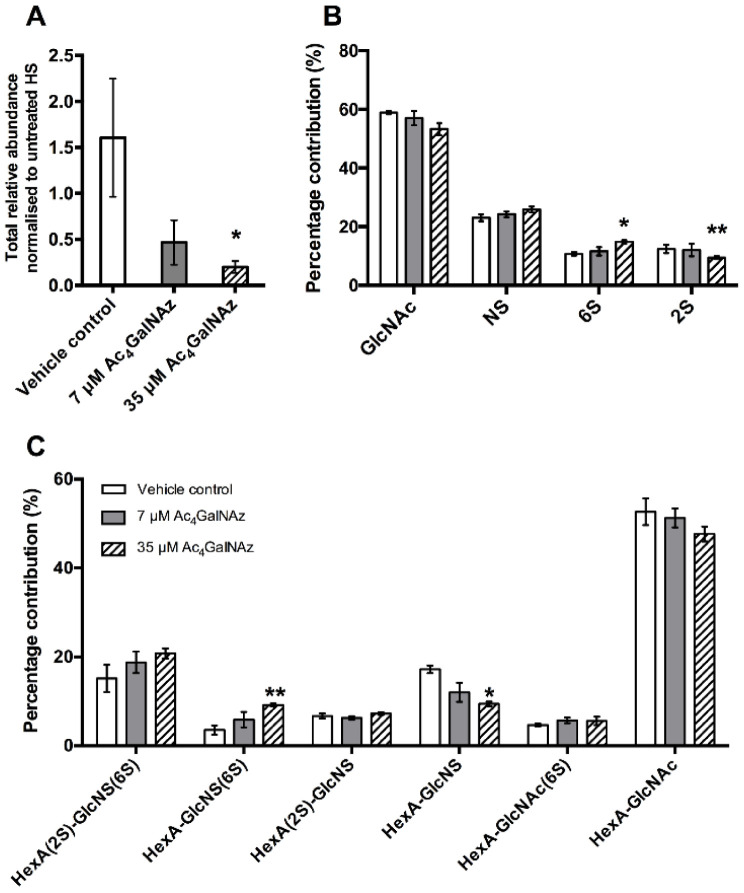
(**A**). Total relative abundance of HS from cell extracted samples. (**B**) Percentage chemical modification contribution of HS and (**C**) percentage contribution of HS disaccharide species after RP-HPLC separation of 2-AMAC-tagged HS. Error bars represent SEM of N = 3 independent experiments. * *p* ≤ 0.05, ** *p* ≤ 0.01, student’s *t* test (two tailed). HexA, hexuronic acid (iduronic or glucuronic acid); 2S, 2-O-sulphate; 6S, 6-O-sulphate; NS, N-sulphate.

To sustain the reduction of HS for longer periods, higher concentrations (35 µM) of azido sugar were required. Both 7 µM and 17.5 µM gave partial population decreases in 10E4 staining and removal of Ac_4_GalNAz returned HS expression levels to match untreated cell populations (24 h rescue), indicating that the effect of the azido sugar treatment on HS was transient and reversible. Significantly less HS was present in 35 µM Ac_4_GalNAz conditions compared with vehicle control conditions with HS depletion displaying a significant dose-dependent decrease (Figure 3A).

**Figure 4:**
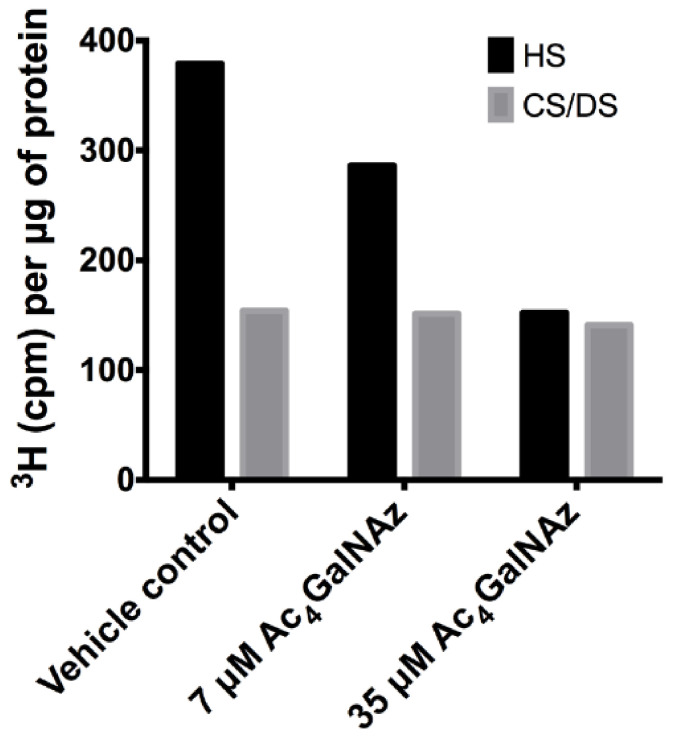
Total GAG synthesis normalised to protein levels from CHO-K1 cells.

Furthermore, HS biosynthesis was perturbed as a subtle, but significant change in disaccharide composition (Figure 3B,C) showing alterations in the sulphation of the chain (increase in 6-O-sulphation and decrease 2-O-sulphation), reminiscent of GAG biosynthetic enzyme mutants [27,28]. To elucidate changes in HS chain length, CHO cell cultures were radiolabelled with ^3^H-glucosamine alongside treatment with Ac_4_GalNAz and HS populations from cell extracts were purified as previously described [29]. Total GAG synthesis was normalised to protein level. Radiolabelled studies showed a dose dependent decrease in the quantity (Figure 4) and chain length of HS (Table 1) in Ac_4_GalNAz-treated cells compared to control.

The marked decrease in chain length observed in both secreted HS and cell-derived HS populations after Ac_4_GalNAz treatment (Table 1) suggests that early termination of chain synthesis was responsible for the depletion of HS at the cell surface observed in flow cytometric experiments (Figure 2).

Despite significant changes to HS chain synthesis, no incorporation of azido groups was detected in HS chains (data not shown), suggesting that either GlcNAz was not incorporated into the chain or that the azide was potentially removed by NDST activity during HS synthesis.

**Figure 5:**
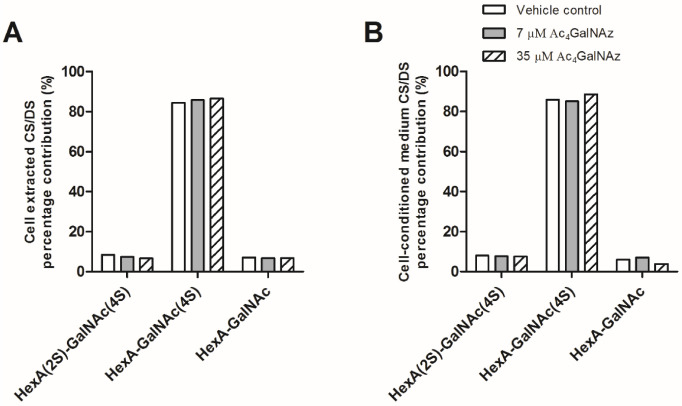
CS/DS disaccharide analysis from (**A**) CHO-K1 cell extracts and (**B**) cell-conditioned medium. Percentage contribution of CS/DS disaccharide species after SAX-HPLC of radiolabelled preparations. Other CS/DS disaccharide species were not detected. HexA, hexuronic acid (iduronic or glucuronic acid). 4S, 4-O-sulphate; 2S, 2-O-sulphate.

Due to convergence in their synthetic pathways (Figure 1) and the utilisation of common precursors, chemical inhibitors usually affect both HS and CS/DS GAGs indiscriminately, therefore we assessed whether CS/DS synthesis was also inhibited by the same metabolic labelling strategy. Azido sugar labelling of CS proteoglycans using GalNAz has been described previously [30,31], but examination of the biosynthesis of CS/DS was not reported. No changes in CS/DS composition (Figure 5), chain length (Table 1) or quantity were observed (Figure 4), suggesting that the inhibitory effect of Ac_4_GalNAz treatment was specific to HS synthesis.

Ac_4_ManNAz has also been reported to label CS proteoglycans, however the presence of CS-specific labelling on the proteoglycan was not investigated [32]. Notably, no NDST enzymes are associated with CS/DS synthesis and the acetyl group of GalNAc remains unmodified, whereas HS biosynthesis specifically involves removal of the acetyl and *N*-sulphation of GlcNAc. This process, at least in part, controls HS chain length [11] possibly via NDST interaction with EXT co-polymerase, although the mechanism is still unclear. EXT enzyme activity in vitro has been demonstrated to utilise UDP-GlcNAz as a substrate [26], suggesting that extension of the HS chain by the EXTs is likely to remain unaffected by GlcNAz *in vivo*. Thus, we hypothesise that the presence of GlcNAz interferes with normal NDST function, thereby inhibiting HS synthesis and resulting in truncated HS chains. Importantly, upregulation of NDST1 has been associated with chemoresistance in breast cancer [33] and upregulated NDST1 activity increases HS biosynthesis [11]. Thus, selective strategies for inhibition of HS activity, such as this one, may have therapeutic potential alongside current treatment options where HS is a known driver of chemoresistance and/or tumour growth.

**Figure 6:**
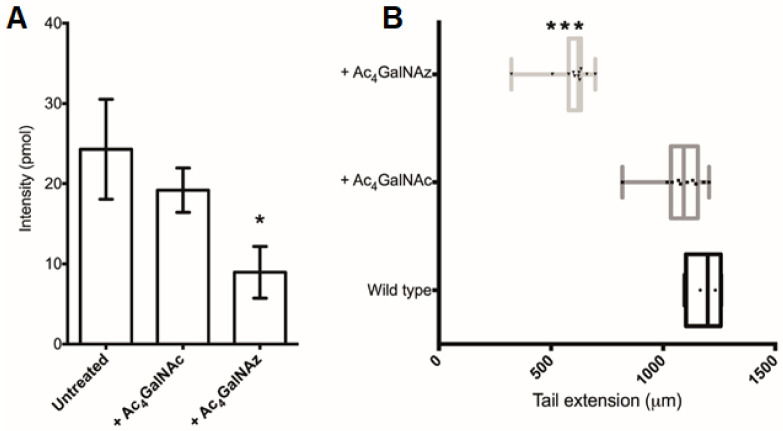
Ac_4_GalNAz treatment of *Xenopus* embryos. (**A**) Total HS (pmol) per embryo. (**B**) Embryo stature measurements of the tail extension (µm). + Ac_4_GalNAz, 500 pmol injection and 500 µM soaking; + Ac_4_GalNAc, 500 pmol injection and 500 µM soaking. * *p* ≤ 0.05, *** *p* ≤ 0.001 + Ac_4_GalNAz vs. Wildtype. Oneway ANOVA with Turkey post-hoc comparison.

Finally, since HS has been also demonstrated to play important roles in development [1], we sought to test the ability of the azido sugar to inhibit HS in a model organism. Therefore, a well-characterised and widely used developmental biology vertebrate model (*Xenopus tropicalis*) was utilised [34]. Following treatment with the inhibitor, the abundance of total HS in Ac_4_GalNAz-treated embryos was decreased compared with controls (Figure 6A), confirming that this small, soluble inhibitor can be used in both organismal and cell culture-based experiments to inhibit HS synthesis.

The Ac_4_GalNAz-treated *Xenopus* embryos displayed a phenotypic short stature (Figure 6B) accompanied with irregular somite boundaries and abnormal skeletal muscle orientation in a dose dependent manner, resulting in gross disorganization of the tail structure and tail kinks (Supplementary Figure S1).

Interestingly, similar developmental abnormalities have been observed in UDP-4-azido-4-deoxyxylose-treated zebrafish [17], where GAG synthesis (CS and HS) was broadly targeted, preventing elongation of either type of GAG. Targeted application of Ac_4_GalNAz as a small, selective HS inhibitor thus provides independent evaluation of the role of HS in biological systems where transient knock down of HS biosynthesis is desired.

## 3. Conclusions

We propose that a common sugar analogue, Ac_4_GalNAz, can be applied as a small, soluble and reversible chemical inhibitor of HS, which does not affect CS/DS biosynthesis, offering a new tool for HS inhibition. Ac_4_GalNAz can be synthesised from inexpensive compounds [35] and is commercially available. Using this strategy, HS inhibition can be achieved in cell-based assays and in whole organisms. The effect of Ac_4_GalNAz on HS production is transient (Figure 2), enabling flexible application and removal in experiments without the need for gene manipulation. This novel selective HS inhibitor therefore may be used to probe HS biology in separation from CS/DS to identify HS-mediated mechanisms in biological systems for further investigation.

## 4. Materials and Methods

### 4.1. Cell Culture, Ac_4_GalNAz and D-[6-^3^H]-Glucosamine Treatment

Chinese Hamster Ovary-K1 (CHO-K1) cells (gifted from the Esko lab) were cultured at 37 °C/5% CO_2_ humidified conditions in Dulbecco’s Modified Eagle Medium: F12 Nutrient Mix (Ham) media (Invitrogen, Loughborough, UK) supplemented with 10% *v*/*v* fetal bovine serum (FBS) (batch-tested, Biosera) and 2 mM L-glutamine (PAA). Cell culture medium was supplemented with sugars dissolved in dimethylsulfoxide (DMSO): Ac_4_GalNAz (Molecular Probes, Loughborough, UK), Ac_4_GalNAc (gifted from the Flitsch group). For radioactive experiments, CHO medium was supplemented with 50 µCi *D-*[6-^3^H]-glucosamine hydrochloride (Perkin Elmer, Llantrisant, UK). CHO-K1 cells were seeded at 40,000 cells/cm^2^ and then cultured for 48 h for metabolic incorporation of the radiolabelled sugar.

### 4.2. Flow Cytometry

To preserve the cell surface, non-enzymatic cell dissociation buffer (Gibco, Loughborough, UK) was used to remove CHOs from tissue culture plastic. After washing with phosphate buffered saline (PBS), cells were incubated with anti-HS (F58–10E4) (1:200, Amsbio, Abingdon, UK) in 0.2% (*w*/*v*) bovine serum albumin (BSA), followed by AlexaFluor goat anti-mouse IgM (κ)-488 (1:1000, Molecular Probes, Loughborough, UK). Cells were fixed with 1% PFA for 10 min at room temperature before analysis on a Cyan ADP cytometer (Beckman Coulter, High Wycombe, UK) using CellQuest Pro software).

### 4.3. GAG Collection and Purification

Cell membranes were dispersed with 1% Triton X-100 in PBS with gentle agitation for 1–2 h. Proteins were digested with 100 µg/mL Pronase (*Streptomyces griseus*, Roche, Welwyn Garden City, UK) for 4 h at 37 °C. Diethylaminoethyl (DEAE) anion-exchange chromatography for GAG preparations with step elution of HS and CS/DS using 1.5 M NaCl was used to isolate GAG material as previously described [36], with the exception of the radiolabelled GAG preparations where gradient anion exchange chromatography (0–1.5 M NaCl) was used. GAG samples were desalted using PD10 columns (GE Healthcare, Amersham, UK) and lyophilised.

### 4.4. HS and CS Chain Length Analysis

Purified *D-*[6-^3^H]-radiolabelled HS or CS material was treated with 50 mM NaOH/1 M NaBH_4_ at 45 °C for 48 h to cleave the protein stub from the xylose residue. Samples were neutralized with glacial acetic acid and then separated on Sepharose CL-6B columns in 0.2 M ammonium bicarbonate at a flow rate of 0.2 mL/min. 1 mL fractions were collected in pony vials (Sigma, Gillingham, UK) and 2 mL Optimax scintillation fluid (Perkin Elmer, Llantrisant, UK) was added. Samples were sealed and shaken before processing for ^3^H radioactivity (counts per minutes) using a liquid scintillation counter (Wallac 1409, Beckman Coulter, High Wycombe, UK). Modal chain length was estimated by comparison of K_av_ values with a calibration curve [37].

### 4.5. Generation of GAG Disaccharide Species

Purified GAG samples were digested either with 2 mIU of each heparinase I–III (Iduron) in 0.1 M sodium acetate, 0.1 mM calcium acetate, pH 7.0 or with chondroitinase ABC (Amsbio, Abingdon, UK) in 50 mM tris, 50 mM NaCl, pH 7.9 for 16 h at 37 °C. For radiolabelled preparations, the disaccharides were separated from oligosaccharides via Superdex-30 chromatography.

### 4.6. 2-AMAC Labelling and RP-HPLC Separation of HS Disaccharides

HS disaccharides were labelled with 2-aminoacridone (2-AMAC) and separated using RP-HPLC as previously described using correction factors for the batch of 2-AMAC utilised [38,39]. Data was also used to calculate the HS sulphation modification.

### 4.7. Strong Anion Exchange (SAX)-HPLC Separation of CS Disaccharides

Samples of 5000 cpm digested *D-*[6-^3^H]-CS disaccharides were separated on a Hypersil 5 µm SAX column (Thermo Scientific, Loughborough, UK) with a gradient of 0.15 M–0.7 M NaCl pH 3.5 over 47 min at a flow rate of 1 mL/min.

### 4.8. Sugar Microinjection and Incubation of Xenopus Embryos

Fertilised NF stage 1 embryos in injection buffer (1% (*w*/*v*) Ficoll in 0.1× Modified Marc Ringers, (MMR), pH 8.0) were injected with 1–5 nL of 500–1000 picomoles Ac_4_GalNAz or Ac_4_GalNAc (dissolved in 0.2 mM KCl) into the cytoplasm using a heat-pulled borosilicate glass capillary injection needle (1 mm × 0.78 mm, Harvard apparatus, Holliston, MA, USA). Embryos were left to recover in injection buffer for 1–2 h (stage 7–8) at 28 °C before they were transferred to fresh agarose-coated dishes containing a bath of 0–500 µM Ac_4_GalNAz or Ac_4_GalNAc in 0.01 × MMR solution. Embryos were incubated at 23 °C (prior to gastrulation) for the first day of development, then at 25 °C and transferred to fresh sugar/0.01 × MMR conditions daily.

### 4.9. Purification of Xenopus HS

Embryos were lyophilised and ground in a pestle and mortar with 1 mL of PBS before addition of 1 mg/mL Pronase in 50 mM Tris/HCl pH 8.0, 1 mM CaCl_2_, 1% Triton X-100. Proteins were digested for 16 h at 55 °C, then a further 0.5 mg Pronase was added and the digestion continued overnight. Pronase was heat-inactivated at 100 °C for 10 min and samples were then treated with 2 µL of 2 M MgCl_2_ and 0.5 µL Benzonase Nuclease (300 mU, Sigma, Gillingham, UK) at 37 °C for 3 h before adjustment to 0.5 M NaOH and mixing overnight. Formic acid was used to adjust the pH to 5.0 prior to centrifugation at 13,000 rpm. The supernatant was diluted with HPLC grade water and applied to DEAE anion exchange chromatography as described in [36] with the following alterations: DEAE beads were washed only with HPLC grade water prior to sample application and samples were eluted with 1 M NaCl, 20 mM NaOAc pH 6.0. The eluent was desalted using PD10 columns according to the manufacturer’s instructions.

### 4.10. Mass Spectrometry Analysis of Xenopus HS Disaccharides

*Xenopus* HS disaccharides were diluted in 200 µL HPLC grade water and centrifuged at 12,000 rpm for 10 min to remove insoluble material. Residual salts and/or proteins were removed from the supernatant using size-exclusion chromatography (Beckman SEC offline fractionate), followed by further clean up using a porous graphite carbon C-18 TopTip (Glygen) prior to Liquid Chormatography-Mass Spectrometry using a Dionex GlycanPac AXH-1 (1 mm × 15 cm) (ThermoFisher, Walton, MA, USA) on an Agilent QTOF 6520 in negative mode, with an acquisition range of 100–1700 *m*/*z*. Heparin disaccharide I-P sodium salt (ΔUA2S-GlcNCOEt6S) (V-labs, Dextra Laboratories, Reading, UK) was spiked into all samples as an external standard to monitor the spray conditions and used for normalization between samples.

### 4.11. Whole Mount Antibody Fluorescent Imaging

Embryos were fixed in 0.1 M 3-(N-Morpholino)propane sulfonic acid (MOPS) pH 7.4, 2 mM ethylene glycol-bis(β-aminoethyl ether)-N,N,N′,N′-tetraacetic acid (EGTA), 1 mM MgSO_4_, 3.7% (*v*/*v*) formaldehyde for 16 h at 4 °C before dehydration with 100% methanol. Embryos were then rehydrated by gradient dilution of the methanol with H_2_O and antibody staining was performed as previously described [40] using mouse 12/101 IgG_1_ (1:200, Developmental Studies Hybridoma Bank) followed by AlexaFluor goat anti-mouse IgG (H + L)-594 (1:500, Molecular Probes). Embryos were imaged using a glass-bottomed dish (MatTek Corporation, Bratislava, Slovakia) and imaged by confocal microscopy using an Olympus Fluoview FV1000 (Olympus, Southend-on-Sea, UK).

## Figures and Tables

**Table 1:** Chain length of radiolabelled HS and CS/DS.

Condition	Secreted Modal Size (kDa)		Cell Extract Modal Size (kDa)	
	HS	CS/DS	HS	CS/DS
Vehicle control	22	32	8.5	38
7 µM Ac_4_GalNAz	12	22	6.9	31
35 µM Ac_4_GalNAz	7	32	7.5	32

## Supplementary Materials

The following are available online at https://www.mdpi.com/article/10.3390/ijms22136988/s1, Figure S1: Wholemount fluorescence of skeletal muscle in stage 39 NF *Xenopus tropicalis* tadpoles after the injection of sugars.
