# Supplementary material for: Selective Inhibition of Heparan Sulphate and Not Chondroitin Sulphate Biosynthesis by a Small, Soluble Competitive Inhibitor
Source: Int J Mol Sci. 2021 Jun 29;22(13):6988. doi: 10.3390/ijms22136988 (PMC8269443; doi:10.3390/ijms22136988)
Supplement: Supplementary file 1 [file ijms-22-06988-s001.zip › ijms-1252281-supplementary.pdf]

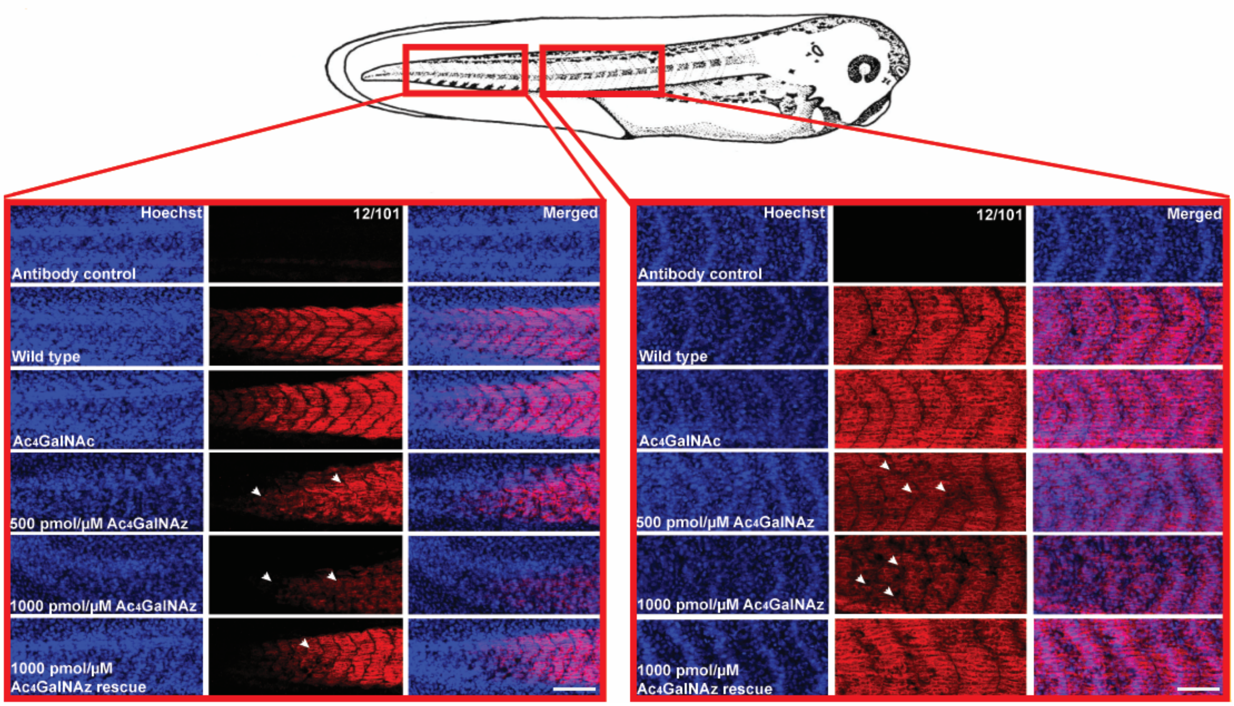

Supplementary Figure S1. Wholemount fluorescence of skeletal muscle in stage 39 NF *Xenopus tropicalis* tadpoles after the injection of sugars. Fertilized eggs (stage 1) were injected with 0-1000 pmol $Ac_4GalNAc$ or $Ac_4GalNAz$ and soaked in 0-1000 uM sugar until developmental stage 39, with daily replacement of the sugar soaking solution. For rescue conditions, $Ac_4GalNAz$ was removed for the final 24 hours. Tadpoles were fixed and blocked and stained with 12/101, followed by AlexFluor-594 to visualize skeletal muscle fibres. Embryos were analysed whole using Z stacking and are presented as flattened 3D images from two independent experiments. White arrows indicate defects in musculature. Blue, Hoechst; red, 12/101. Scale bar, 50 μm.
